# Vegetarian diet is associated with a lower risk of chronic kidney disease in a population-based study

**DOI:** 10.1038/s41598-026-62827-2

**Published:** 2026-08-03

**Authors:** Catharina J. Candussi, William Bell, Marko Mutapcic, Alysha S. Thompson, Sabine Rohrmann, Aedín Cassidy, Tilman Kühn, Martina Gaggl

**Affiliations:** 1 Department of Nutritional Sciences, University of Vienna, Vienna, Austria; 2 Center for Public Health, Public Health Nutrition, Medical University of Vienna, Vienna, Austria; 3 Vienna Doctoral School of Pharmaceutical, Nutritional and Sport Sciences, University of Vienna, Vienna, Austria; 4 Co-Centre for Sustainable Food Systems and the Institute for Global Food Security, Queen’s University Belfast, Belfast, Northern Ireland, UK; 5 Division of Chronic Disease Epidemiology, Epidemiology, Biostatistics and Prevention Institute (EBPI), University of Zurich, Zurich, Switzerland; 6 Cancer Registry of the Cantons Zurich, Zug, Schaffhausen and Schwyz, University Hospital Zurich, Zurich, Switzerland; 7 Department of Nutritional, Food and Consumer Sciences, Fulda University of Applied Sciences, Fulda, Germany; 8 Cancer Epidemiology Unit, Nuffield Department for Population Health, University of Oxford, Oxford, UK

**Keywords:** Chronic kidney disease, CKD, Plant-based diet, Vegetarian, Vegan, Diseases, Health care, Medical research, Nephrology, Risk factors

## Abstract

The global prevalence of chronic kidney disease (CKD) is rising and initial studies suggest that diets predominantly based on greater inclusion of plant foods may be associated with lower CKD risk. As population-based studies are lacking, we investigated the association between habitual plant-based diets and CKD in the UK Biobank cohort. The UK Biobank is a large prospective cohort study of participants aged 40–69 years. Habitual diet was assessed using a baseline food frequency questionnaire, and participants were classified into five dietary groups: high meat eaters, low meat eaters, poultry eaters, pescatarians, and vegetarians. To assess the risk of CKD across these groups, we conducted multivariable Cox proportional hazards regression analyses. During follow up of 12.9 years 23,084 out of 416,584 developed CKD. Compared to high meat eaters, the strongest association was observed among vegetarians which had a statistically significant lower risk of CKD [HR = 0.81, 95% CI: 0.71– 0.93]. Our population-based study on plant-based diet types and CKD risk suggests that vegetarian diets are associated with a lower risk of CKD. Future research is needed to assess the feasibility and acceptability of plant-based diets for the prevention of CKD and other chronic diseases.

**Supplementary Information:**

The online version contains supplementary material available 10.1038/s41598-026-62827-2.

## Introduction

Plant-based diets have received substantial attention in recent years, partly due to evidence linking high consumption of animal-based foods with adverse health outcomes^1–3^. Moreover, plant-based diets are associated with both better cardiometabolic health and a lower environmental impact^4,5^. The prevalence of chronic kidney disease (CKD) is increasing worldwide, yet evidence on the potential role of plant-based diets in this vulnerable patient population is limited. In particular, data on the role of habitual dietary patterns in CKD prevention remain scarce. Several studies using dietary indices, such as the healthy plant-based diet index, suggest that individuals with reduced kidney function may benefit from a healthy, balanced diet similar to that recommended for the general population^6–8^. Initial evidence also indicates a lower incidence of CKD among those consuming diets rich in high-quality plant foods and low in animal products^9^. However, these studies typically assessed diet using post hoc diet quality scores, and there is a lack of data examining habitual diet types in relation to CKD. While diet quality indices have consistently shown associations with CKD risk, they rely on investigator-defined scoring systems and may not fully capture real-world dietary behaviors. By contrast, habitual diet groups represent naturally occurring dietary patterns and may therefore provide complementary and more directly translatable evidence for clinical and public health recommendations.

Therefore, the aim of this study was to assess the associations between habitual diet groups - high meat eaters, low meat eaters, poultry eaters, fish eaters, vegetarians and vegans - and incident CKD using data from the population-based UK Biobank study. Our hypothesis was that a habitual diet with a greater emphasis on plant-based foods would be associated with a lower incidence of CKD, consistent with their known cardiometabolic benefits in the general population.

## Materials & methods

We used data from the UK Biobank, a large prospective cohort comprising over 500,000 participants aged 40–69 years at the time of recruitment. Baseline data collection was carried out between 2006 and 2010 at 22 assessment centres across Wales, Scotland and England. At baseline recruitment, participants completed a comprehensive touchscreen questionnaire that gathered information on socioeconomic status, lifestyle factors and health conditions. Dietary intake was assessed using a food frequency questionnaire (FFQ), capturing the consumption of 29 specific food items. In addition, biological material and physical measurements were collected by trained personnel. Further information on the study design, recruitment process, and baseline assessments has been documented elsewhere^10,11^. The UK Biobank study received ethical approval from the National Health Service North West Multi-Centre Research Ethics Committee. The initial approval was granted in 2011 under the reference number 11/NW/0382. Subsequent renewals have occurred every five years, with approvals granted in 2016 (16/NW/0274) and 2021 (21/NW/0157)^12,13^. All participants provided written informed consent and all methods were performed in accordance with the relevant guidelines and regulations.

For the current analysis, a complete-case approach was applied. Of the 502,166 included participants, individuals with missing dietary frequency data at baseline (*n* = 7,748) were excluded, resulting in 494,418 participants with available dietary data. Participants with missing continuous covariates were further excluded, including missing BMI (*n* = 2,586), waist circumference (*n* = 104), eGFR (*n* = 53,986), and ACR data (*n* = 12,101), resulting in 425,641 participants with complete datasets. After excluding participants with prevalent CKD at baseline (*n* = 9,057), the final analytical cohort consisted of 416,584 participants. Additionally, descriptive statistics of nutritional intakes (Supplementary Table 6) of only 178,209 participants were analyzed due to incomplete dietary data on 24-hour recall (Supplementary Fig. 1).

### Dietary assessment and categorization

Similar to Parra-Soto *et al.* and Bradbury *et al.* we classified our study participants into six different dietary groups (high meat eaters, low meat eaters, poultry eaters, pescatarians, vegetarians, and vegans) based on their responses to a touchscreen FFQ administered at baseline^14,15^. From 0 (never) to 5 (once or more per day), the questionnaire assessed the consumption of 29 food items—such as various types of meat (e.g., processed meat, lamb, pork, beef, chicken, turkey), fish (oily and non-oily), eggs, and cheese.

When reporting to eat red or processed meat (including poultry) more than 5–6 times per week participants were classified as high meat eaters. Low meat eaters consumed these items less frequently, but more than once a week (Supplementary Table 1). Poultry eaters reported eating poultry but not red or processed meat. Pescatarians excluded red, processed meat, and poultry but included fish in their diet. Vegetarians consumed no meat or fish, while vegans reported never consuming any animal-derived foods^14,15^. However, after the initial classification, we found that the number of participants adhering to a vegan diet was too small to allow for statistically robust analyses. To address this limitation, we merged, as previously done by Tong *et al.* vegetarians and vegans into a single group, referred to as vegetarians^16^. Additionally, data on average nutrient intakes (e.g., source of protein, fiber, potassium, etc.) was derived from the validated Oxford WebQ 24-hour dietary recall questionnaire^17^.

### Case ascertainment

CKD diagnoses were identified using the International Classification of Diseases, 10th Revision (ICD-10) codes (N03, N06, N08, N11–N16, N18, N19, Z49, I12, I13) or the Office of Population Censuses and Surveys Classification of Interventions and Procedures, version 4 (OPCS-4), from hospital inpatient data (L74.1–L74.6, L74.8, L74.9, M01.2, M01.3–M01.5, M01.8, M01.9, M02.3, M08.4, M17.2, M17.4, M17.8, M17.9, X40.1–X40.9, X41.1, X41.2, X41.8, X41.9, X42.1, X42.8, X42.9, and X43.1).Full details can be found in Supplementary Table 2.

We classified CKD as prevalent diagnosis if (1) a diagnosis occurred prior to the initial dietary assessment, based on self-reported information and hospital inpatient records, or (2) participants had a baseline estimated glomerular filtration rate (eGFR) below 60 mL/min/1.73 m².

### Covariate assessment

The following list of covariates was assessed and obtained at baseline recruitment: sex (categorical: Male; Female), age (continuous: Years), type 2 diabetes diagnosis (categorical: Yes; No), ethnicity (categorical: Asian, Black, Mixed, White, Unknown), income before tax (categorical: Less than 18,000, 18,000 to 30,999, 31,000 to 51,999, 52,000 to 100,000, Greater than 100,000, Unknown), education (categorical: High, Medium, Low, Unknown), smoking status (categorical: Never, Previous, Current), alcohol frequency (categorical: Prefer not to answer, Daily or almost daily, Three or four times a week, once or twice a week, One to three times a month, Special occasions only, Never), Body Mass Index (BMI) (continuous: kg/m^2^), waist circumference (continuous: cm), physical activity (categorical: High, Moderate, Low, Unknown), albumin-creatinine ratio (ACR) (categorical: >300 mg/g, 300 − 30 mg/g, < 30 mg/g), eGFR (continuous: ml/min per 1.73m^2^, polygenic risk (High, Medium, Low). The eGFR was calculated using the R package kidney.epi, which utilizes the creatinine-based CKD-EPI (Chronic Kidney Disease Epidemiology Collaboration) 2021 equation^18^. The ACR was calculated as follows^19^.

$$\:ACR\left(mg/g\right)=\frac{Urinary\:albumin\left(mg/L\right)}{Urinary\:creatinine\:(\mu\:mol/L)\times\:\frac{0.11312}{1000}}$$

As proposed by the Kidney Disease Improving Global Outcomes (KDIGO) we divided CKD into 5 stages (Supplementary Table 3). The polygenic risk for eGFR was adapted as proposed by Thompson *et al.* whereby a high polygenic risk score for eGFR reflects a low polygenic risk for CKD^20^. Accordingly, participants were classified into three groups based on tertiles of the polygenic eGFR score: high genetic risk for CKD (polygenic eGFR score − 0.028 to 0.0569), intermediate genetic risk for CKD (> 0.0569 to 0.0768), and low genetic risk for CKD (> 0.0768 to 0.1549). Higher polygenic eGFR scores indicate a greater genetic predisposition to higher kidney function and therefore a lower genetic risk of CKD. A table of all covariates and their respective coding and item number from the UK Biobank’s online showcase can be found in Supplementary Table 4.

### Statistical analysis

Descriptive statistics were examined across five dietary groups (high meat eaters, low meat eaters, poultry eaters, pescatarians, vegetarians). Continuous variables were expressed as means with standard deviations (± SD), while categorical variables were summarized as percentages. Associations between dietary patterns and incidence of CKD were assessed using Cox proportional hazards regression models, with age as the underlying time scale, implemented via the R packages survival and survminer^21,22^.

Covariates were selected based on a comprehensive review of the literature on established and potential risk factors for CKD, informed by studies such as Charles *et al*.^23^, Cheung *et al*.^24^ and Wang *et al.*^25^. Primary analyses were adjusted for sociodemographic factors (sex, ethnicity, income after tax, education), lifestyle factors (smoking status, alcohol frequency, physical activity), and clinical measures (Type 2 diabetes (T2DM), BMI, waist circumference). Subgroup analyses were conducted stratified by (i) kidney function at baseline (normal vs*.* CKD stage 1–2), (ii) sex (female vs*.* male), (iii) polygenic risk (low vs. medium vs*.* high), (iv) BMI (< 25 vs. ≥ 25), and (v) education (low, medium, high). In sensitivity analyses, models were further adjusted for (i) ACR and eGFR, and (ii) a polygenic risk. We adjusted for eGFR and ACR in sensitivity analyses to account for basal variation that may be associated with prospective CKD risk.

Hazard ratios (HRs) and 95% confidence intervals (CIs) were estimated using Cox proportional hazards models. The proportional hazards assumption was assessed with Schoenfeld residuals and was not violated for the main exposures. Since the assumption was not met for ethnicity and T2DM, these variables were stratified in the models. To evaluate heterogeneity across HRs, Cochrane’s Q and corresponding p-values were calculated using the R package metafor^26^.

A two-sided p-value of less than 0.05 was considered indicative of statistical significance. All analyses were conducted using R (version 4.3.1)^27^.

## Results

### Study population

Over a mean (±SD) follow-up period of 12.9 (±2.8) years, 23,084 participants developed CKD among them 90% had normal kidney function at baseline, while 3% and 7% had CKD stage 1 and 2, respectively (Supplementary Table 5). Individuals who developed CKD had a higher mean BMI compared to those who did not (29.0 ± 5.2 vs. 27.2 ± 4.7). Moreover, 27% of participants with T2DM developed CKD, compared to 7% of those without a history of T2DM (Supplementary Table 5). Within our study population, 195,030 participants were high meat eaters, 199,478 low meat eaters, 4,779 poultry eaters, 9,647 pescatarians, and 7,650 vegetarians.

When comparing dietary groups, individuals following a vegetarian diet appeared more likely to be female, younger, have a lower BMI, a higher baseline eGFR, and a higher educational attainment compared to those adhering to a high meat eaters (Table 1).

Nutrient intake varied descriptively across dietary groups. High meat eaters had the highest intake of animal-based protein, whereas vegetarians had the highest intake of plant-based protein. Vegetarians also appeared to consumed more fiber but less potassium and phosphate compared to high meat eaters (Supplementary Table 6). Overall, meat eaters had higher urinary excretion of sodium, creatinine, and microalbumin than non–meat eaters (Supplementary Table 7). No formal statistical tests were conducted for these descriptive comparisons.

**Table 1:** Descriptive statistics among diet groups.

Characteristic	High meat eater *N* = 195,030	Low meat eater *N* = 199,478	Poultry eater *N* = 4,779	Pescatarian *N* = 9,647	Vegetarian *N* = 7,650
Sex					
Female	84,005 (43%)	126,364 (63%)	3,652 (76%)	6,924 (72%)	5,082 (66%)
Male	111,025 (57%)	73,114 (37%)	1,127 (24%)	2,723 (28%)	2,568 (34%)
Age	56.46 (8.13)	56.61 (7.97)	56.45 (8.03)	54.13 (7.99)	53.12 (7.89)
Incident cases	11,796 (6%)	10,516 (5%)	221 (5%)	319 (3%)	232 (3%)
T2DM diagnosis	18,445 (9%)	14,116 (7%)	251 (5%)	359 (4%)	510 (7%)
Ethnicity					
White	186,254 (96%)	188,951 (95%)	4,322 (90%)	9,015 (93%)	5,850 (80%)
Asian	2,934 (2%)	3,949 (2%)	205 (4%)	285 (3%)	1,235 (16%)
Black	2,665 (1%)	3,002 (2%)	130 (3%)	138 (1%)	42 (1%)
Mixed	1,075 (1%)	1,186 (1%)	31 (1%)	87 (1%)	62 (1%)
Unknown	2,102 (1%)	2,390 (1%)	91 (2%)	122 (1%)	124 (2%)
Average income before tax					
Less than 18,000	36,442 (19%)	37,887 (19%)	1,174 (25%)	1,655 (17%)	1,408 (18%)
18,000 to 30,999	42,608 (22%)	43,734 (22%)	983 (21%)	1,981 (21%)	1,512 (20%)
31,000 to 51,999	44,902 (23%)	44,788 (22%)	921 (19%)	2,344 (24%)	1,862 (24%)
52,000 to 100,000	35,328 (18%)	35,063 (18%)	751 (16%)	2,125 (22%)	1,522 (20%)
Greater than 100,000	9,283 (5%)	9,615 (5%)	189 (4%)	513 (5%)	305 (4%)
Unknown	26,467 (14%)	28,391 (14%)	761 (16%)	1,029 (11%)	1,041 (14%)
Education					
High	88,744 (46%)	96,249 (48%)	2,506 (52%)	6,490 (67%)	4,753 (62%)
Medium	37,798 (19%)	35,023 (18%)	792 (17%)	1,352 (14%)	1,209 (16%)
Low	66,613 (34%)	66,326 (33%)	1,412 (30%)	1,739 (18%)	1,586 (21%)
Unknown	1,875 (1%)	1,880 (1%)	69 (1%)	66 (1%)	102 (1%)
Smoking status					
Current	23,746 (12%)	17,660 (9%)	373 (8%)	677 (7%)	508 (7%)
Previous	68,385 (35%)	68,266 (34%)	1,565 (33%)	3,479 (36%)	2,263 (30%)
Never	102,266 (52%)	112,874 (57%)	2,824 (59%)	5,463 (57%)	4,853 (63%)
Unknown	633 (0%)	678 (0%)	17 (0%)	28 (0%)	26 (0%)
Alcohol frequency					
Prefer not to answer	130 (0%)	125 (0%)	1 (0%)	6 (0%)	9 (0%)
Daily or almost daily	46,247 (24%)	36,507 (18%)	656 (14%)	1,838 (19%)	1,051 (14%)
Three or four times a week	48,645 (25%)	44,866 (22%)	843 (18%)	2,285 (24%)	1,341 (18%)
Once or twice a week	49,514 (25%)	53,579 (27%)	1,114 (23%)	2,203 (23%)	1,530 (20%)
One to three times a month	19,417 (10%)	24,292 (12%)	531 (11%)	1,117 (12%)	888 (12%)
Special occasions only	18,750 (10%)	24,522 (12%)	833 (17%)	1,132 (12%)	1,086 (14%)
Never	12,327 (6%)	15,587 (8%)	801 (17%)	1,066 (11%)	1,745 (23%)
BMI	27.87 (4.76)	27.02 (4.60)	25.50 (4.52)	25.25 (4.30)	25.62 (4.58)
Waist circumference	92.62 (13.29)	88.18 (12.89)	83.02 (12.40)	83.19 (12.08)	85.03 (12.75)
Physical activity					
High	47,384 (24%)	48,330 (24%)	1,457 (30%)	2,684 (28%)	1,968 (26%)
Moderate	76,137 (39%)	80,539 (40%)	1,806 (38%)	4,237 (44%)	3,204 (42%)
Low	28,746 (15%)	27,241 (14%)	518 (11%)	1,040 (11%)	997 (13%)
Unknown	42,763 (22%)	43,368 (22%)	998 (21%)	1,686 (17%)	1,481 (19%)
ACR					
< 30 mg/g	186,480 (96%)	191,668 (96%)	4,581 (96%)	9,298 (96%)	7,358 (96%)
> 300 mg/g	587 (0%)	471 (0%)	16 (0%)	23 (0%)	17 (0%)
300 − 30 mg/g	7,963 (4%)	7,339 (4%)	182 (4%)	326 (3%)	275 (4%)
eGFR	95.24 (13.30)	95.88 (13.18)	97.31 (13.36)	99.78 (12.72)	98.77 (13.30)
Polygenic risk					
High	55,100 (33%)	55,091 (33%)	1,239 (34%)	2,525 (33%)	1,829 (34%)
Medium	54,737 (33%)	55,494 (33%)	1,223 (34%)	2,566 (34%)	1,763 (33%)
Low	55,114 (33%)	55,211 (33%)	1,163 (32%)	2,529 (33%)	1,766 (33%)
Unknown	30,079	33,682	1,154	2,027	2,292
CKD stages					
Normal	186,480 (96%)	191,668 (96%)	4,581 (96%)	9,298 (96%)	7,358 (96%)
Stage 1	4,944 (3%)	4,767 (2%)	118 (2%)	235 (2%)	191 (2%)
Stage 2	3,606 (2%)	3,043 (2%)	80 (2%)	114 (1%)	101 (1%)

*BMI* body mass index (calculated as weight in kilograms divided by height in meters squared; kg/m2), *eGFR* estimated glomerular filtration rate (mL/min per 1.73m^2^), *ACR* urinary albumin to creatinine ratio (mg/g); waist circumference (cm)

Categorical variables are expressed as the number of cases, followed by the percentage in parentheses: n (%). Continuous variables are expressed as the arithmetic mean and standard deviation (mean ± (SD)).

### Diet group and risk of CKD

**Fig. 1:**
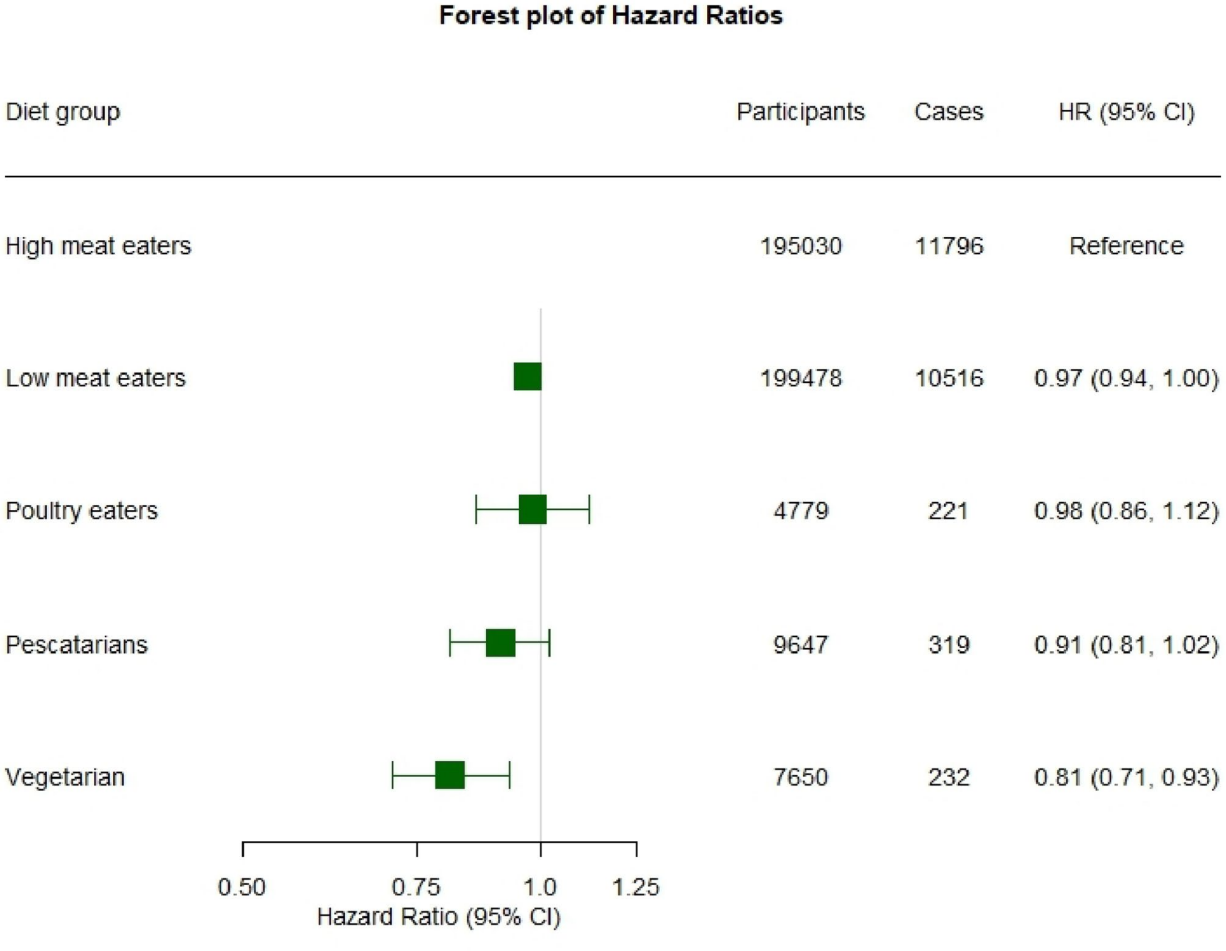
Hazard ratios (HR) and 95% Confidence intervals (95% CI) between diet groups and the risk of chronic kidney disease (CKD). Age was included as the underlying time variable. Models adjusted for sex, income and education level, waist circumference, body mass index (BMI), physical activity and smoking status and stratified by ethnicity and type 2 diabetes mellitus (T2DM) diagnosis.

In fully adjusted models (Fig. 1), low meat eaters [HR = 0.97, 95% CI: 0.943–0.996] and vegetarians [HR = 0.81, 95% CI: 0.711–0.927] had a significantly lower risk of CKD compared with high meat eaters.

Stratification by baseline kidney function showed that vegetarians with normal kidney function had a lower risk of CKD [HR = 0.81, 95% CI: 0.706–0.936], while no significant association was observed among those with mildly reduced kidney function (CKD stages 1–2); however, there was no evidence of heterogeneity (*p* = 0.27–0.84, Supplementary Fig. 2). The inverse association for vegetarian diets was slightly more pronounced in men (21% lower risk, 95% CI: 0.64–0.98) than in women (16% lower risk, 95% CI: 0.71–0.99), but no significant heterogeneity by sex was observed (Supplementary Fig. 3). Participants within the lowest polygenic risk group (indicative of higher kidney function) appeared to benefit most, with a ~ 30% lower risk of CKD compared with high meat eaters, although the associations were consistent across polygenic risk strata and no heterogeneity was detected (*p* = 0.35 to 0.95) (Supplementary Fig. 4). In subgroup analyses across different BMI and educational groups, varying trends were observed. However, tests for heterogeneity did not reach statistical significance (Supplementary Fig. 5–6).

In sensitivity analyses with additional adjustment for baseline eGFR and ACR, the association observed among vegetarians was slightly strengthened [HR = 0.80, 95% CI: 0.676–0.938] (Supplementary Fig. 7). After further adjustment for polygenic risk, the association for pescatarians became stronger and reached statistical significance [HR = 0.89, 95% CI: 0.75–0.97], while the association for vegetarians remained similar [HR = 0.79, 95% CI: 0.67–0.94] (Supplementary Fig. 8).

To assess whether the observed associations were influenced by differences in ethnic composition across dietary groups, we conducted a sensitivity analysis stratified by ethnicity. The direction and magnitude of the associations were broadly consistent across ethnic groups, and no significant heterogeneity was observed for any dietary pattern (all p-heterogeneity > 0.05). In particular, the lower CKD risk observed among vegetarians was evident across ethnic groups, although estimates among Asian and Black participants were less precise due to smaller sample sizes (Supplementary Fig. 9).

## Discussion

In this large population-based study, we observed a gradient in CKD risk reduction across low meat eaters (3% lower), poultry eaters (2% lower), pescatarians (9% lower) and vegetarians (19% lower) compared to high meat eaters. These associations paralleled a gradient in animal protein intake and were consistent across population subgroups, suggesting that vegetarian diets may provide the greatest protection against CKD.

While diets rich in fruits and vegetables confer health benefits in the general population^8,28,29^, recommendations for individuals with CKD can be contradictory. Protein restriction, used to slow CKD progression, favors diets low in animal products, whereas potassium-restricted diets to prevent hyperkalemia limit many plant-based foods. Additionally, dietary phosphate restriction—key to managing CKD-related mineral and bone disorders (CKD-MBD) —often leads to monotonous diets with limited variety.

Our results are in line with findings from a US cohort study, which categorised 14,686 participants into overall, healthy, and less healthy plant-based diet indices, as well as a pro-vegetarian diet index. Over a median follow-up of 24 years, almost 30% developed incident CKD, with risk 10–14% lower among those adhering to a pro-vegetarian diet and healthy plant-based diets. Of note, CKD incidence was considerably higher in *this population* than in the UK Biobank, likely reflecting differences in follow-up length, cohort composition, and baseline risk^9^. Similarly, in a Taiwanese cohort with self-reported diet groups, individuals following plant-based diets had lower CKD risk^30^. Prior studies using dietary pattern and diet quality scores have consistently shown that greater inclusion of plant-based foods is associated with lower incidence of CKD^31^, slower disease progression, and reduced mortality among CKD patients^6,32,33^. Interestingly, we show that, there was no evidence of heterogeneity across strata of genetic risk, indicating that the association between vegetarian dietary patterns and lower CKD risk was broadly consistent regardless of genetic predisposition.

The biological mechanisms through which vegetarian diets might mitigate the development of kidney disease are not elucidated in detail. However, three main mechanisms might positively influence the development and progression of CKD, respectively.

1. Vegetarian diets are usually low in organic phosphate compounds, organic sulfate, and non-metabolizable organic acids, which lead to proton excess and subsequent acid loading^34,35^. Experimental studies have shown that renal tissue acidification enhances the local renin-angiotensin system and that angiotensin II, aldosterone and endothelin promote renal fibrosis, leading to progression of CKD^35,36^. Clinical studies in patients with hypertensive nephropathy showed that a diet rich in fruits and vegetables reduced net acid excretion and halted the progression of CKD, when compared to drug-based intervention (oral sodium bicarbonate)^37–39^.
2. Vegetarian diets come with a higher dietary intake of fiber and polyphenols. Polyphenols have previously been associated with a lower risk of CKD and a reduced all-cause mortality in patients with CKD^40,41^. Higher dietary fiber intake supports a healthier gut microbiome and is associated with a lower incidence of CKD^42–44^ and mortality^45^, possibly due to an increase in short-chain fatty acid (SCFA)-producing bacteria, which have been shown to reduce oxidative stress and inflammation^46^. Vegetarian diet may also lower circulating trimethylamine-N-oxide (TMAO) levels, a gut-derived metabolite associated with higher mortality and cardiometabolic risk in CKD^47^- thus, TMAO may be a target for therapeutic strategies in CKD^48–50^. In addition, comorbidities strongly associated with CKD, such as diabetes or hypertension, are less common with higher fiber intake^51^. Recently, it has been shown that dietary fiber intake reduces the development of diabetic nephropathy *via *an increased number of SCFA-producing bacteria^52^, and halts the progression of CKD-MBD in mouse models^53^. The gut-kidney axis appears to play a pivotal role in the development of CKD and associated comorbidities^54^, and thus diet represents a modifiable risk factor to prevent these diseases and mitigate their progression.
3. Micronutrients are present in reasonable concentrations in plant-based meals. While effects of minerals such as potassium and phosphate on kidney function have been analysed in detail, the effects of other micronutrients are less well understood. In the *Tehran Lipid and Glucose Study (TLGS*), the risk of incident CKD was lower with higher intakes of vitamins C, E, D, cobalamin, and folate^55^. This partly contrasts with the findings of the *Ansan-Ansung cohort* of *the Korean Genome and Epidemiologic Study (KoGES*), in which participants with higher vitamin C and B6 intakes had a higher risk of developing CKD over a 12-year follow-up period^56^. However, beneficial micronutrient intake may reduce oxidative stress and inflammation and subsequently modify the progression of CKD^57^. Interestingly, high potassium intake was associated with a lower incidence of CKD in a recent *UK Biobank *analysis^58^. Compared with individuals in the lowest quintile of dietary potassium intake, those in the highest quintile had a 14% lower risk of incident CKD. This association was even more pronounced when urinary potassium-to-creatinine excretion was used as a surrogate marker of potassium intake. High quality plant-based diets are high in potassium and low in sodium. The detrimental effects of high sodium intake are well known, and the effect of a low-sodium diet may also partly explain our findings^59,60^. Similarly, red meat–rich diets are high in phosphate, a key driver of CKD-MBD, which in turn is a major contributor to the excess mortality observed in CKD populations^9^. In addition, higher dietary phosphate intake is also associated with higher mortality in the general population^61^. In plant foods, phosphate occurs mainly as phytate, which is poorly absorbed due to the lack of intestinal phytase, thereby reducing phosphate burden^62–64^.

### Limitations

This study has several limitations. The UK Biobank cohort is characterized by a generally healthier risk profile relative to the UK general population and predominantly composed of participants of white European descent^65^. This may limit the generalizability of the findings to more diverse populations. Another key limitation relates to its observational design, which precludes the exclusion of residual or unmeasured confounding. Notably, differences in baseline characteristics across dietary groups suggest that dietary patterns are embedded within broader lifestyle profiles^66^. Although statistical adjustments were performed, residual confounding remains a concern. Consequently, the observed associations - particularly the modest effect size observed for vegetarians - may partly reflect overall healthier lifestyle patterns rather than diet alone, and causal interpretations should therefore be made with caution.

An additional limitation concerns the classification of dietary groups, which combines frequency-based and exclusion-based criteria. For example, “high” and “low” meat eaters are defined by consumption frequency, whereas poultry eaters and pescatarians are defined by the exclusion of specific food groups. This inconsistency complicates interpretation, as individuals consuming poultry or fish frequently may be classified into groups that are implicitly interpreted as more plant-based. As a result, the ability to interpret findings along a clear dose–response gradient of animal protein intake is limited, and some degree of exposure misclassification cannot be ruled out.

With regard to outcome assessment, CKD was identified using hospital records and diagnostic codes, which are more likely to capture clinically recognized or advanced cases rather than all incident CKD cases. Furthermore, for subgroup analyses in participants with CKD stage 1–2 and participants with normal kidney function, we utilized the KDIGO guidelines for CKD staging. However, baseline eGFR and ACR measurements were assessed at a single time point only, which deviates from the clinical definition of CKD and CKD staging that requires persistent abnormalities over at least three months. Consequently, some participants classified as having early kidney impairment may have had transient reductions in kidney function or temporary albuminuria rather than persistent CKD. Although a considerable number of CKD cases were recorded during follow-up, the statistical power to detect moderate associations among vegans was limited due to the relatively small size of this subgroup (*n*= 367). However, vegan and vegetarian diets have partially distinct nutritional profiles, which may lead to a dilution of associations when combining them^67^. Lastly, participants may have changed their dietary habits over time or underreported certain food items, which may lead to dietary misclassification, which in fact may have led to an underestimation of the true risk estimates. However, a reproducibility study in a subsample of the UK Biobank suggests that diet types remain very stable over time in this cohort^68^.

## Conclusion

Our study showed that vegetarian diets are associated with a lower risk of CKD. This finding is consistent with previous research highlighting the benefits of plant-based dietary patterns, as captured by various dietary indices^6,7,32^. Notably, recent clinical guidelines such as those from KDIGO (Kidney Disease: Improving Global Outcomes) have begun to acknowledge and support the role of plant-based diets in CKD management^69^. However, in clinical practice, more conservative approaches - often based on outdated concerns regarding potassium, phosphorus, or protein quality - still prevail^70^. Given the potential benefit of plant-based diets in the prevention of CKD, randomized controlled trials are needed on plant-based diets and prognosis among CKD patients, including assessing their practicability and acceptability.

## Supplementary Information

Below is the link to the electronic supplementary material.

Supplementary Material 1

## Data Availability

This research was conducted using data from UK Biobank under application number 64226. The data are not publicly available but can be accessed by bona fide researchers upon application to UK Biobank (www.ukbiobank.ac.uk).

## Declarations

### **Generative AI and AI-assisted technologies in the manuscript preparation process**

generative AI and AI-assisted technologies in the manuscript preparation process readability of the text. After using this tool/service, the author(s) reviewed and edited the content as needed and take(s) full responsibility for the content of the published article.
