# Supplementary material for: Vegetarian diet is associated with a lower risk of chronic kidney disease in a population-based study
Source: Sci Rep. 2026 Aug 3;16:23849. doi: 10.1038/s41598-026-62827-2 (PMC13434791; doi:10.1038/s41598-026-62827-2)
Supplement: Supplementary file 1 [file 41598_2026_62827_MOESM1_ESM.pdf]

Supplemental material:

Vegetarian diet is associated with a lower risk of chronic kidney disease in a population-based study, Candussi et al.

Supplementary Figure 1: *Flow chart of included participants*

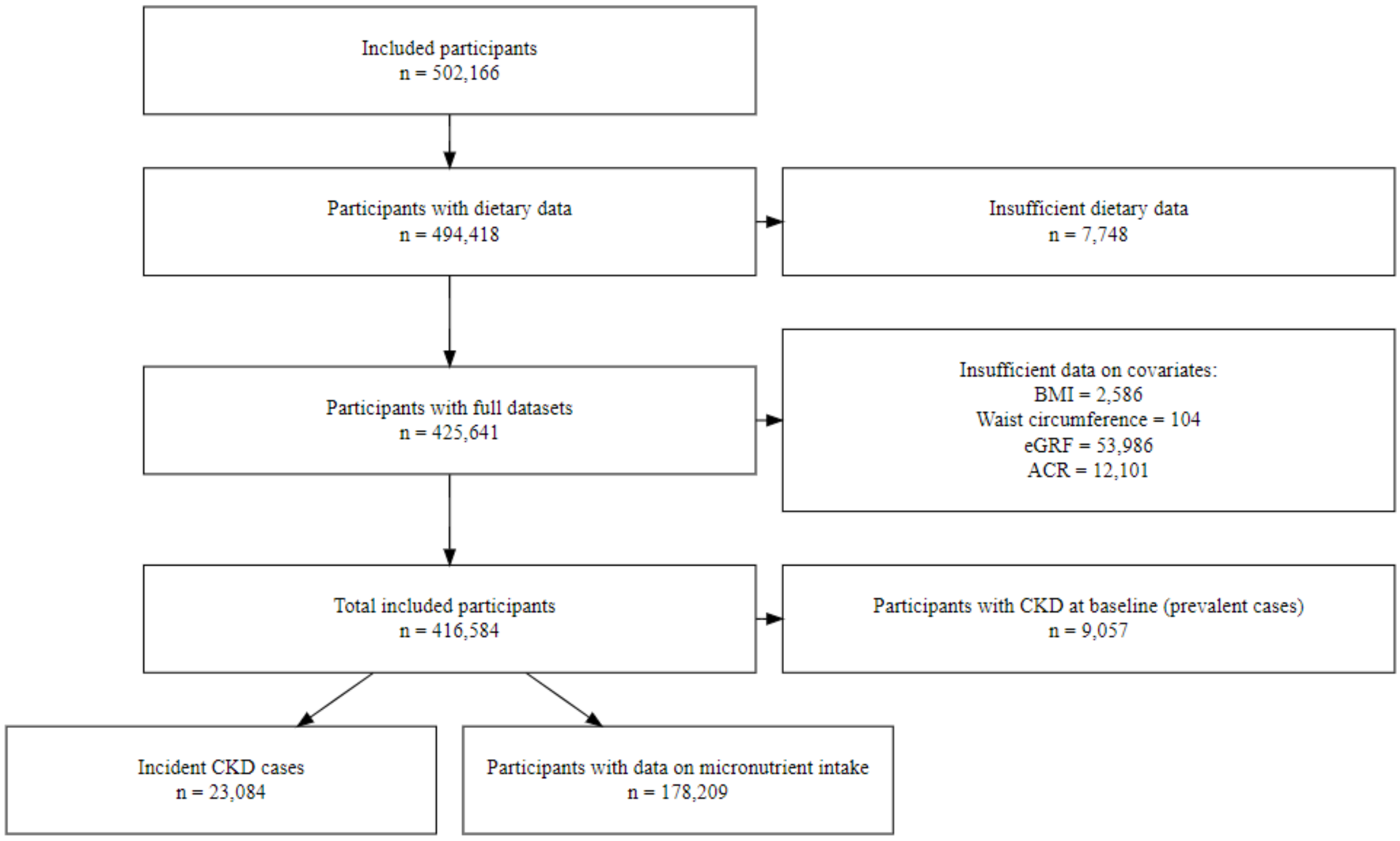

Supplementary Table 1*: Diet classification*

	High meat eater	Low meat eater	Poultry eater	Pescatarian	Vegetarian
Processed meat	More than 5 times per week	1 – 4 times per week			
Beef					
Pork					
Lamb, mutton					
Chicken, turkey, poultry					
Fish					
Egg					
Dairy					

Supplementary Table 2: *Cases assessment of chronic kidney disease (CKD)*

Code Type	Code	UK Biobank Showcase Data Field
Self-reported non-cancer illness	1192, 1193, 1194, 1607	20002
ICD-9 codes	585.9	41271
ICD-10 codes	N03, N06, N08, N11, N12, N13, N14, N15, N16, N18, N19, Z49, I12, I13	41270
OPCS-3 codes	560.2, 561, 561.1, 562, 563.1, 563.2, 563.3, 564, 564.1, 565, 566, 566.1, 567.1, 567.2, 568, 569, 571.1	41273
OPCS-4 codes	L74.1, L74.2, L74.3, L74.4, L74.5, L74.6, L74.8, L74.9, M01.2, M01.3, M01.4, M01.5, M01.8, M01.9, M02.3, M08.4, M17.2, M17.4, M17.8, M17.9, X40.1, X40.2, X40.3, X40.4, X40.5, X40.6, X40.7, X40.8, X40.9, X41.1, X41.2, X41.8, X41.9, X42.1, X42.8, X42.9, X43.1	41272

Supplementary Table 3: *CKD Stage classification adapted from KDIGO (Kidney Disease: Improving Global Outcomes)*[1]

CKD Stage	eGFR	ACR
Normal kidney function	> 90	<= 30
Stage 1	> 90	> 30
Stage 2	>= 60 & <= 90	> 30
Stage 3	>= 30 & < 60	
Stage 4	>= 15 & < 30	
Stage 5	< 15	

Abbreviation: eGFR, estimated glomerular filtration rate (mL/min per 1.73m2); ACR, urinary albumin to creatinine ratio (mg/g);

Supplementary Table 4: *Information on categorization of covariates*

Variables	UK Biobank Showcase Data Field	Type	Coding
Sex	31	Categorical: Male, Female	NA
Age	21022	Continuous: Years	NA
T2DM Diagnosis	1712	Categorical: Yes, No	ICD10 E11 Codes
Ethnicity	100065	Categorical: Asian, Black, Mixed, White, Unknown	NA
Income before tax	738	Categorical: „Less than 18,000“, „18,000 to 30,999“ , „31,000 to 51,999“, “52,000 to 100,000”, “Greater than 100,000”, “Missing”	NA
Education	6138	Categorical: Low, Medium, High, Unknown	Low = “None of the above”, “O-level/GCSE”, “CSE or equivalent”<br><br>Medium = “A-level/AS Level”, “NVQ, HND, HNC”<br><br>High = “College or University Degree”, “Other Professional Qualification”<br><br>Unknown = „prefer not to answer“, missing, „none of the above“
Smoking status	20116	Categorical: Never, Previous, Current, Unknown	NA
Alcohol frequency	1558	Categorical: Prefer not to answer, Daily or almost daily, Three or four times a week, Once or twice a week, One to three times a month, Special occasions only, Never	NA
BMI (Body Mass Index)	23104	Continuous: kg/m2	NA
Waist circumference	48	Continuous: cm	NA
Physical activity	874, 894, 914	Categorical: High, Moderate, Low, Unknown	Low = under 600 minutes/week<br>Moderate = 600-3000 minutes/week<br>High = over 3000 minutes/week
ACR (albumin creatinine ratio)	30510, 30505	Categorical: <30 mg/g, 30-300 mg/g, >300 mg/g	
eGFR (estimated glomerular filtration rate)	23478, 21000	Continuous: $mL/min/1.73m^2$	Calculated using creatinine-based CKD-EPI 2021 from the R package kidney.epi [2]
Polygenic risk		Categorical: High, Medium, Low	Polygenic risk score was calculated based on Thompson et al. and subsequently divided into tertiles[3]

Supplementary Table 5: *Descriptive characteristics among incident CKD cases and non-cases*

		**Non- cases**<br>N = 393,500	**Cases**<br>N = 23,084	**Overall**<br>N = 416,584
Diet	High meat eater	183,234 (47%)	11,796 (51%)	195,030 (47%)
	Low meat eater	188,962 (48%)	10,516 (46%)	199,478 (48%)
	Poultry eater	4,558 (1%)	221 (1%)	4,779 (1%)
	Pescatarian	9,328 (2%)	319 (1%)	9,647 (2%)
	Vegetarian	7,418 (2%)	232 (1%)	7,650 (2%)
Sex	Female	215,029 (55%)	10,998 (48%)	226,027 (54%)
	Male	178,471 (45%)	12,086 (52%)	190,557 (46%)
Age		56.14 (8.06)	61.02 (6.72)	56.41 (8.07)
T2DM diagnosis		27,570 (7%)	6,111 (26%)	33,681 (8%)
Ethnicity	White	372,875 (95%)	21,854 (95%)	394,729 (95%)
	Asian	8,152 (2%)	456 (2%)	8,608 (2%)
	Black	5,581 (1%)	396 (2%)	5,977 (1%)
	Mixed	2,336 (1%)	105 (0%)	2,441 (1%)
	Unknown	4,556 (1%)	273 (1%)	4,829 (1%)
Average income before tax	Less than 18,000	71,678 (18%)	6,888 (30%)	78,566 (19%)
	18,000 to 30,999	85,396 (22%)	5,422 (23%)	90,818 (22%)
	31,000 to 51,999	90,872 (23%)	3,945 (17%)	94,817 (23%)
	52,000 to 100,000	72,525 (18%)	2,264 (10%)	74,789 (18%)
	Greater than 100,000	19,433 (5%)	472 (2%)	19,905 (5%)
	Unknown	53,596 (14%)	4,093 (18%)	57,689 (14%)
Education	High	190,188 (48%)	8,554 (37%)	198,742 (48%)
	Medium	72,226 (18%)	3,948 (17%)	76,174 (18%)
	Low	127,386 (32%)	10,290 (45%)	137,676 (33%)
	Unknown	3,700 (1%)	292 (1%)	3,992 (1%)
Smoking status	Current	40,338 (10%)	2,626 (11%)	42,964 (10%)
	Previous	134,371 (34%)	9,587 (42%)	143,958 (35%)
	Never	217,519 (55%)	10,761 (47%)	228,280 (55%)
	Unknown	1,272 (0%)	110 (0%)	1,382 (0%)

		**Non- cases**<br>N = 393,500	**Cases**<br>N = 23,084	**Overall**<br>N = 416,584
Alcohol frequency	Prefer not to answer	253 (0%)	18 (0%)	271 (0%)
	Daily or almost daily	81,870 (21%)	4,429 (19%)	86,299 (21%)
	Three or four times a week	93,682 (24%)	4,298 (19%)	97,980 (24%)
	Once or twice a week	102,306 (26%)	5,634 (24%)	107,940 (26%)
	One to three times a month	43,600 (11%)	2,645 (11%)	46,245 (11%)
	Special occasions only	42,888 (11%)	3,435 (15%)	46,323 (11%)
	Never	28,901 (7%)	2,625 (11%)	31,526 (8%)
BMI		27.23 (4.66)	29.04 (5.18)	27.33 (4.71)
Waist circumference		89.70 (13.21)	95.53 (13.88)	90.02 (13.31)
Physical activity	High	96,632 (25%)	5,191 (22%)	101,823 (24%)
	Moderate	157,639 (40%)	8,284 (36%)	165,923 (40%)
	Low	54,965 (14%)	3,577 (15%)	58,542 (14%)
	Unknown	84,264 (21%)	6,032 (26%)	90,296 (22%)
ACR category	<30 mg/g	378,701 (96%)	20,684 (90%)	399,385 (96%)
	>300 mg/g	770 (0%)	344 (1%)	1,114 (0%)
	300-30 mg/g	14,029 (4%)	2,056 (9%)	16,085 (4%)
eGFR		96.41 (12.90)	84.33 (14.03)	95.74 (13.26)
Polygenic risk	High	108,519 (33%)	7,265 (37%)	115,784 (33%)
	Medium	109,335 (33%)	6,448 (33%)	115,783 (33%)
	Low	109,949 (34%)	5,834 (30%)	115,783 (33%)
	Unknown	65,697	3,537	69,234
CKD stages	Normal	378,701 (96%)	20,684 (90%)	399,385 (96%)
	Stage 1	9,548 (2%)	707 (3%)	10,255 (2%)
	Stage 2	5,251 (1%)	1,693 (7%)	6,944 (2%)

Abbreviation: BMI, body mass index (calculated as weight in kilograms divided by height in meters squared; kg/m2); eGFR, estimated glomerular filtration rate (mL/min per 1.73m2); ACR, urinary albumin to creatinine ratio (mg/g); waist circumference (mm)
Categorical variables are expressed as the number of cases, followed by the percentage in parentheses: n (%). Continuous variables are expressed as the arithmetic mean and standard deviation (mean ± SD).

Supplementary Table 6: *Nutritional intake among different dietary groups*

	**High meat eater** N = 79,948	**Low meat eater** N = 86,976	**Poultry eater** N = 2,091	**Pescatarian** N = 5,215	**Vegetarian** N = 3,979
Protein average (g/day)	85 (24)	78 (22)	72 (23)	68 (20)	62 (19)
Animal protein (g/day)	57 (21)	51 (19)	42 (20)	33 (16)	23 (13)
Plant-based protein (g/day)	28 (9)	27 (10)	30 (12)	35 (13)	40 (15)
Potassium average (mg/day)	3.70 (1.01)	3.60 (1.02)	3.65 (1.15)	3.68 (1.04)	3.62 (1.10)
Sodium average (mg/day)	2.07 (0.78)	1.82 (0.69)	1.73 (0.70)	1.91 (0.70)	1.93 (0.71)
Phosphate average (mg/day)	1.45 (0.37)	1.39 (0.36)	1.37 (0.39)	1.39 (0.37)	1.36 (0.39)
Fibre average (g/day)	17 (6)	18 (6)	20 (8)	21 (7)	22 (8)
Vitamin E average (mg/day)	11.0 (4.3)	10.6 (4.2)	11.5 (4.7)	12.4 (4.6)	12.6 (5.2)
Vitamin D average (µg/day)	3.71 (2.78)	3.56 (2.90)	3.92 (3.58)	3.82 (3.41)	2.05 (1.57)
Vitamin C average (mg/day)	122 (74)	130 (77)	146 (88)	143 (82)	146 (86)

Continuous variables are expressed as the arithmetic mean and standard deviation (mean ± SD).

Supplementary Table 7: *Urinary marker among different dietary groups*

	**High meat eater** N = 194,363	**Low meat eater** N = 198,543	**Poultry eater** N = 4,735	**Pescatarian** N = 9,576	**Vegetarian** N = 7,599
Sodium (mmol/L)	83 (46)	73 (43)	63 (41)	67 (42)	73 (48)
Potassium (mmol/L)	65 (34)	62 (34)	59 (35)	59 (35)	59 (35)
Creatinine (mmol/L)	9.50 (5.88)	8.35 (5.60)	7.32 (5.46)	7.15 (5.24)	7.25 (5.43)
Microalbumin (mg/L)	9 (58)	7 (47)	7 (61)	6 (34)	6 (45)

Continuous variables are expressed as the arithmetic mean and standard deviation (mean ± SD).

Supplementary Figure 2*: Subgroup analysis with hazard ratios (HR) and 95% confidence intervals (95% CI) between diet groups and the risk of chronic kidney disease (CKD) among people with normal kidney function and people having CKD Stage 1 or 2 at baseline. Age included as the underlying time variable. Model adjusted for sex, income and education level, waist circumference, BMI, physical activity and smoking status and stratified by ethnicity and T2DM diagnosis.*

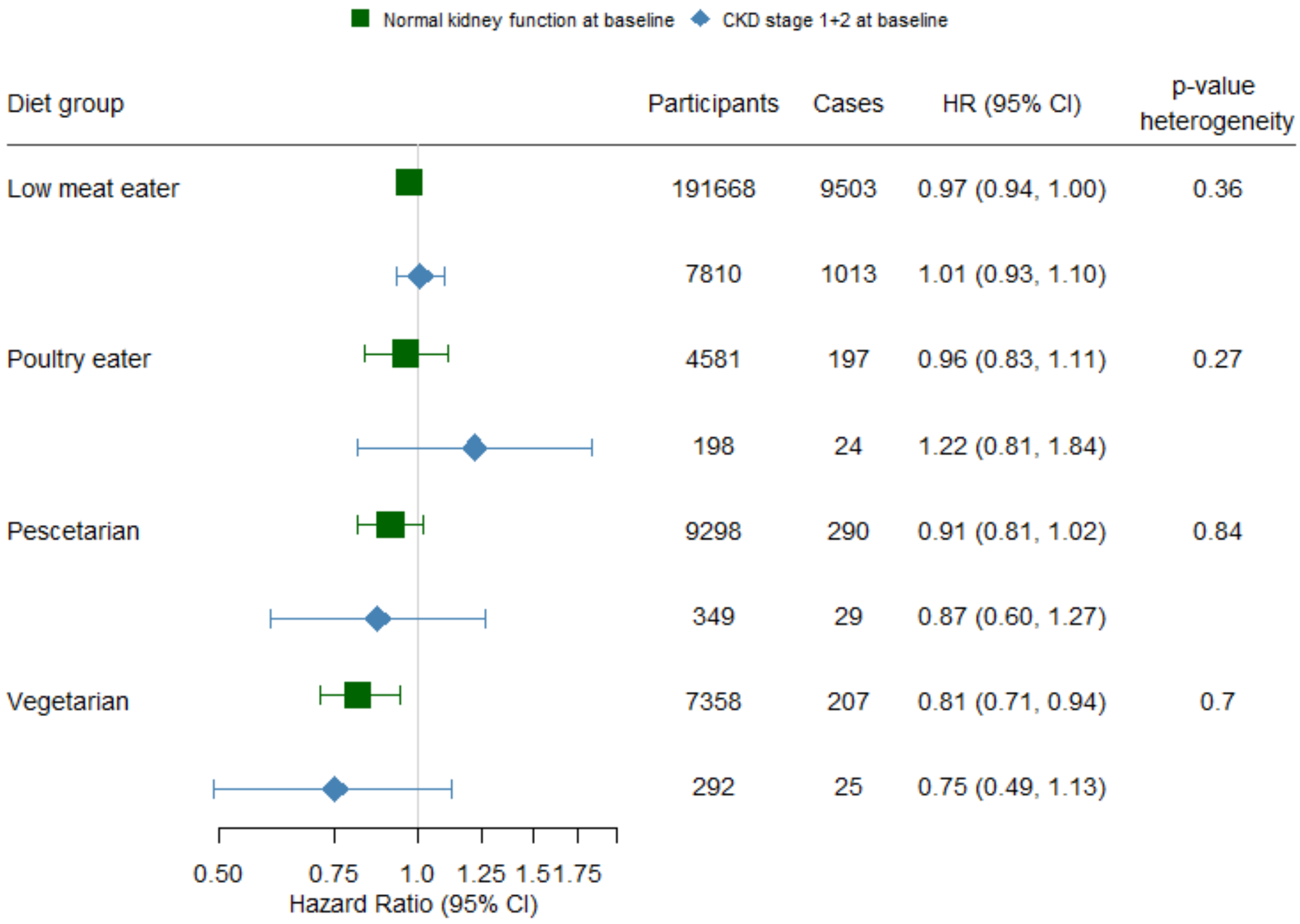

Supplementary Figure 3*: Subgroup analysis with hazard ratios (HR) and 95% confidence intervals (95% CI) between diet groups and the risk of chronic kidney disease (CKD) among females and males. Age included as the underlying time variable. Model adjusted for income and education level, waist circumference, BMI, physical activity and smoking status and stratified by ethnicity and T2DM diagnosis.*

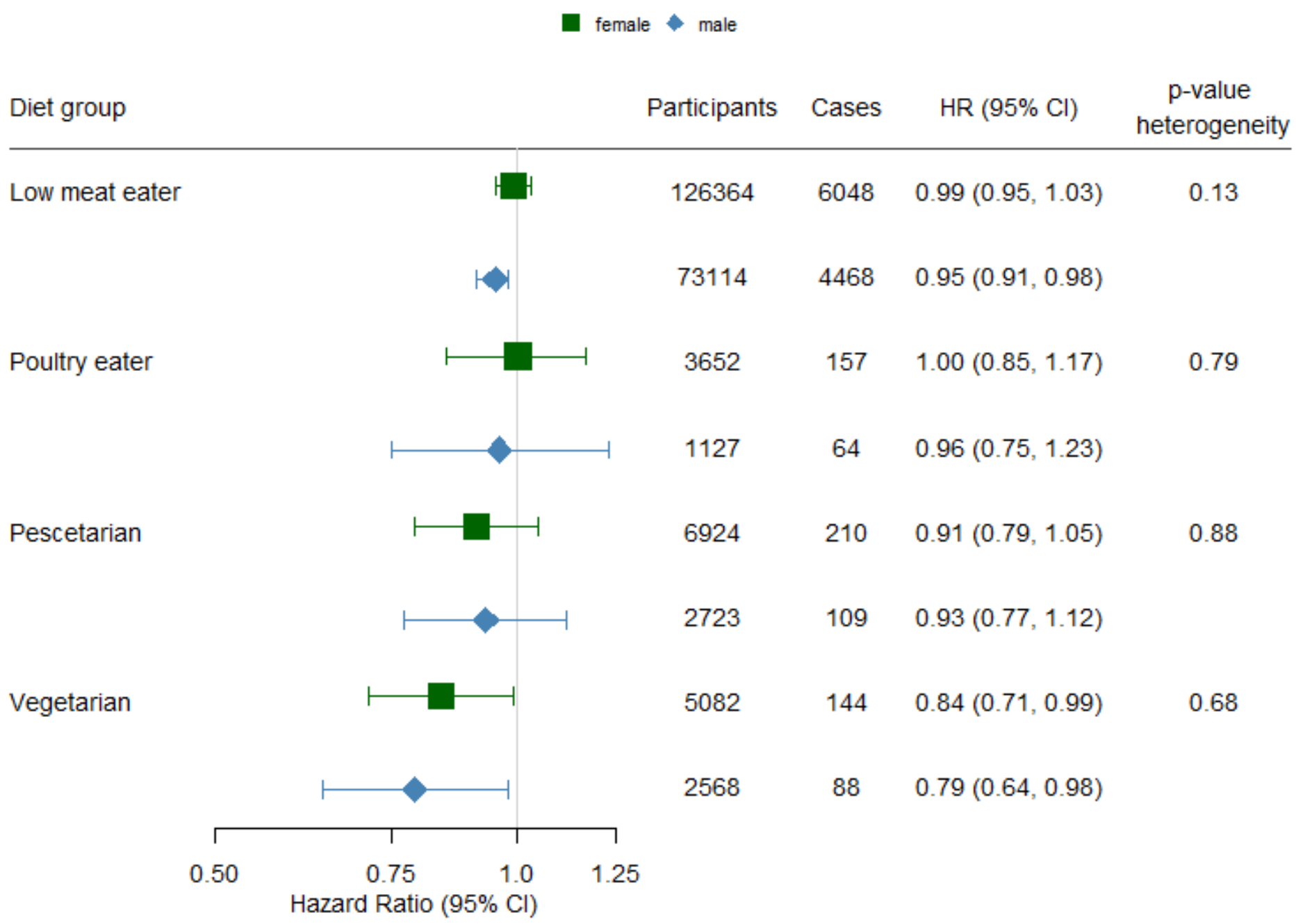

Supplementary Figure 4: *Subgroup analysis with hazard ratios (HR) and 95% confidence intervals (95% CI) between diet groups and the risk of chronic kidney disease (CKD) among people with low, medium and high polygenic risk. Age included as the underlying time variable. Model adjusted for sex, income and education level, waist circumference, BMI, physical activity and smoking status and stratified by ethnicity and T2DM diagnosis.*

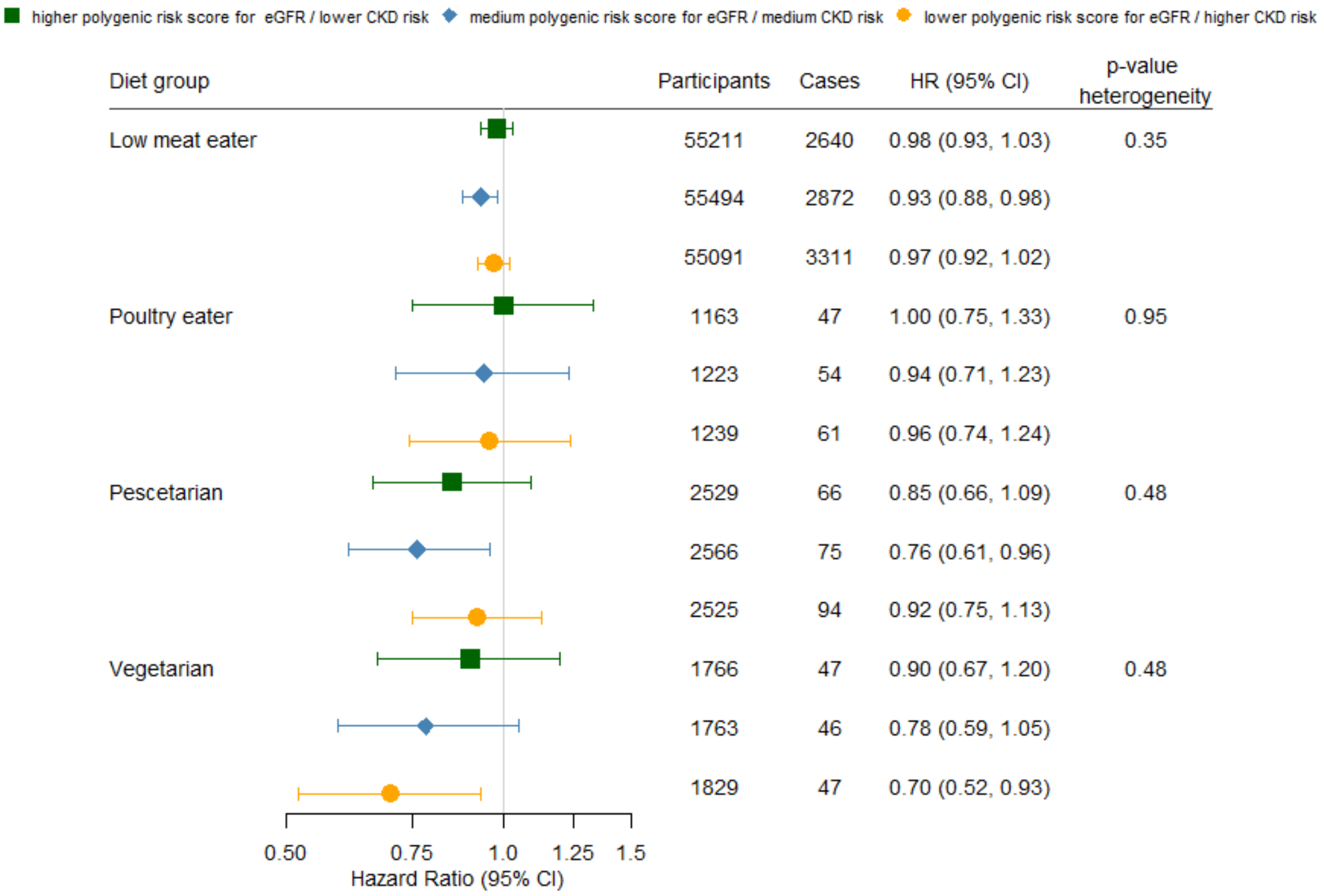

Supplementary Figure 5: *Subgroup analysis with hazard ratios (HR) and 95% confidence intervals (95% CI) between diet groups and the risk of chronic kidney disease (CKD) among individuals with BMI lower or higher than 25. Age included as the underlying time variable. Model adjusted for sex, income and education level, waist circumference, physical activity and smoking status and stratified by ethnicity and T2DM diagnosis.*

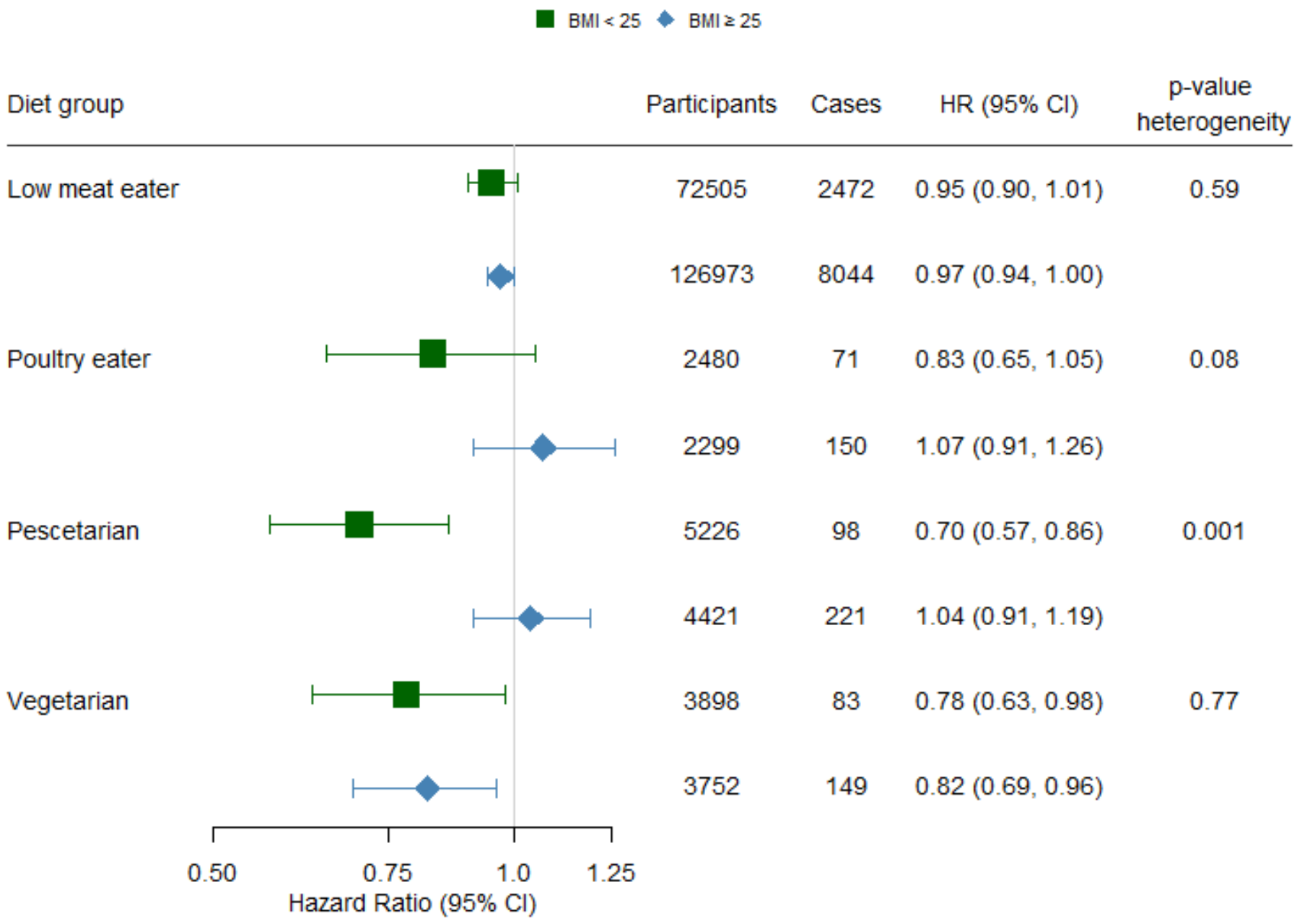

Supplementary Figure 6: *Subgroup analysis with hazard ratios (HR) and 95% confidence intervals (95% CI) between diet groups and the risk of chronic kidney disease (CKD) among individuals with low, medium and high education. Age included as the underlying time variable. Model adjusted for sex, income and education level, waist circumference, physical activity and smoking status and stratified by ethnicity and T2DM diagnosis.*

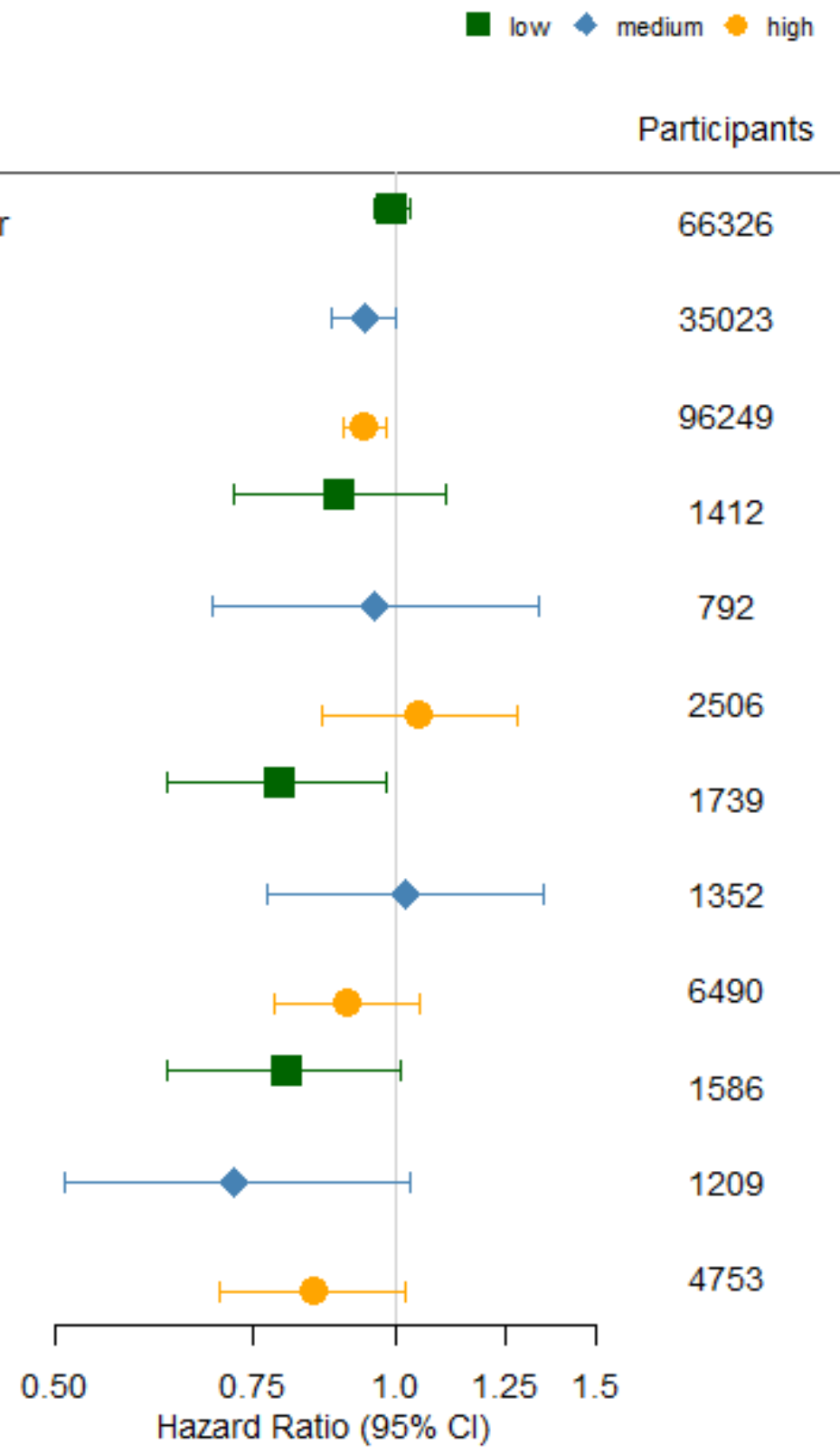

Diet group	Participants	Cases	HR (95% CI)	p-value heterogeneity
Low meat eater	66326	4768	0.99 (0.96, 1.03)	0.14
	35023	1697	0.94 (0.88, 1.00)	
	96249	3925	0.94 (0.90, 0.98)	
Poultry eater	1412	81	0.89 (0.72, 1.11)	0.56
	792	36	0.96 (0.69, 1.34)	
	2506	100	1.05 (0.86, 1.28)	
Pescetarian	1739	79	0.79 (0.63, 0.98)	0.35
	1352	51	1.02 (0.77, 1.35)	
	6490	181	0.91 (0.78, 1.05)	
Vegetarian	1586	77	0.80 (0.63, 1.01)	0.73
	1209	32	0.72 (0.51, 1.03)	
	4753	121	0.85 (0.70, 1.02)	

Supplementary Figure 7*: Sensitivity analysis with hazard ratios (HR) and 95% confidence intervals (95% CI) between diet groups and the risk of chronic kidney disease (CKD). Age included as the underlying time variable. Basemodel adjusted for sex, income and education level, waist circumference, BMI, physical activity and smoking status and stratified by ethnicity and T2DM diagnosis. Basemodel including biomarker additionally adjusted for estimated glomerular filtration rate (eGFR) and urinary albumin to creatinine ratio (ACR).*

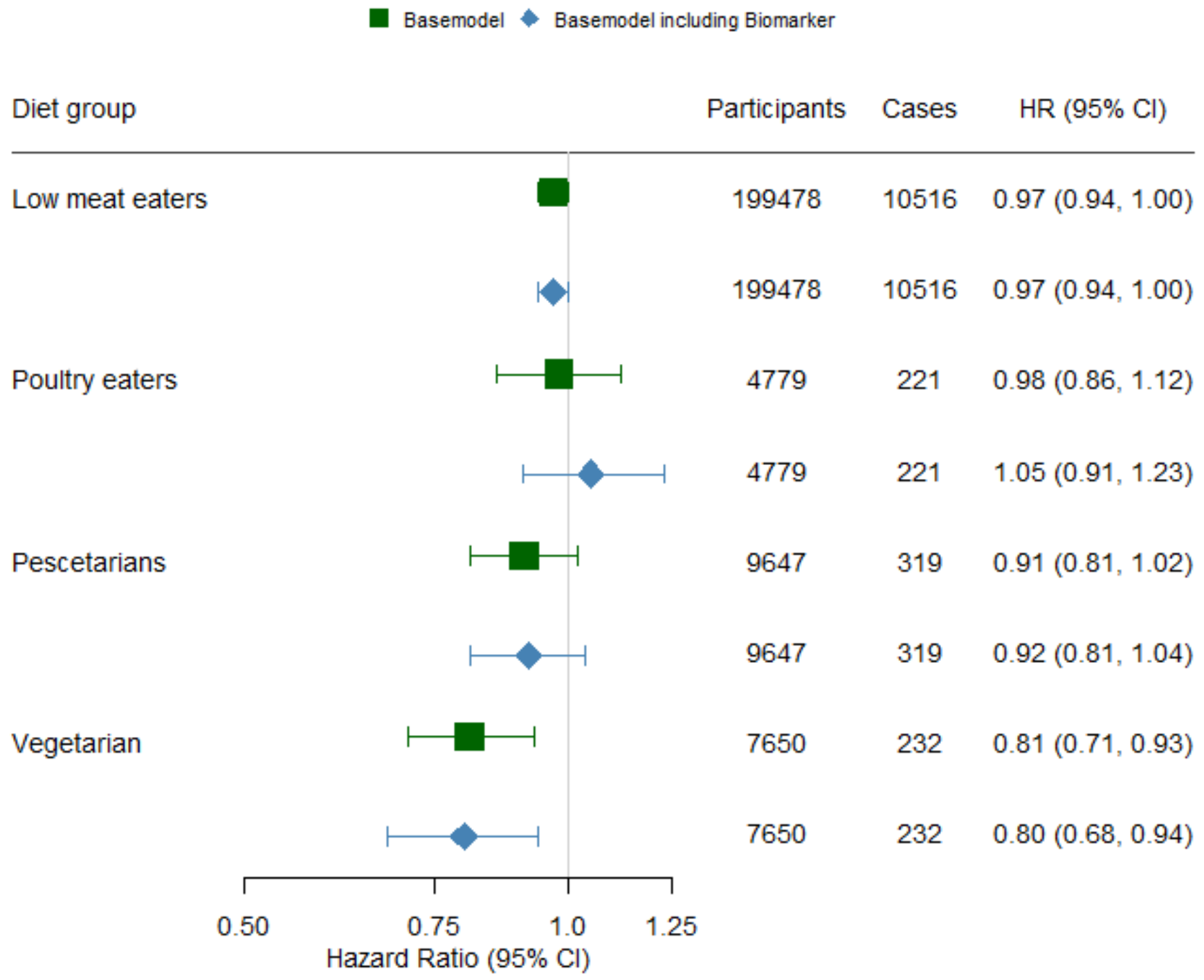

Supplementary Figure 8: Sensitivity *analysis with hazard ratios (HR) and 95% confidence intervals (95% CI) between diet groups and the risk of chronic kidney disease (CKD). Age included as the underlying time variable. Basemodel adjusted for sex, income and education level, waist circumference, BMI, physical activity and smoking status and stratified by ethnicity and T2DM diagnosis. Basemodel including polygenic risk (PR) additionally adjusted for polygenic risk.*

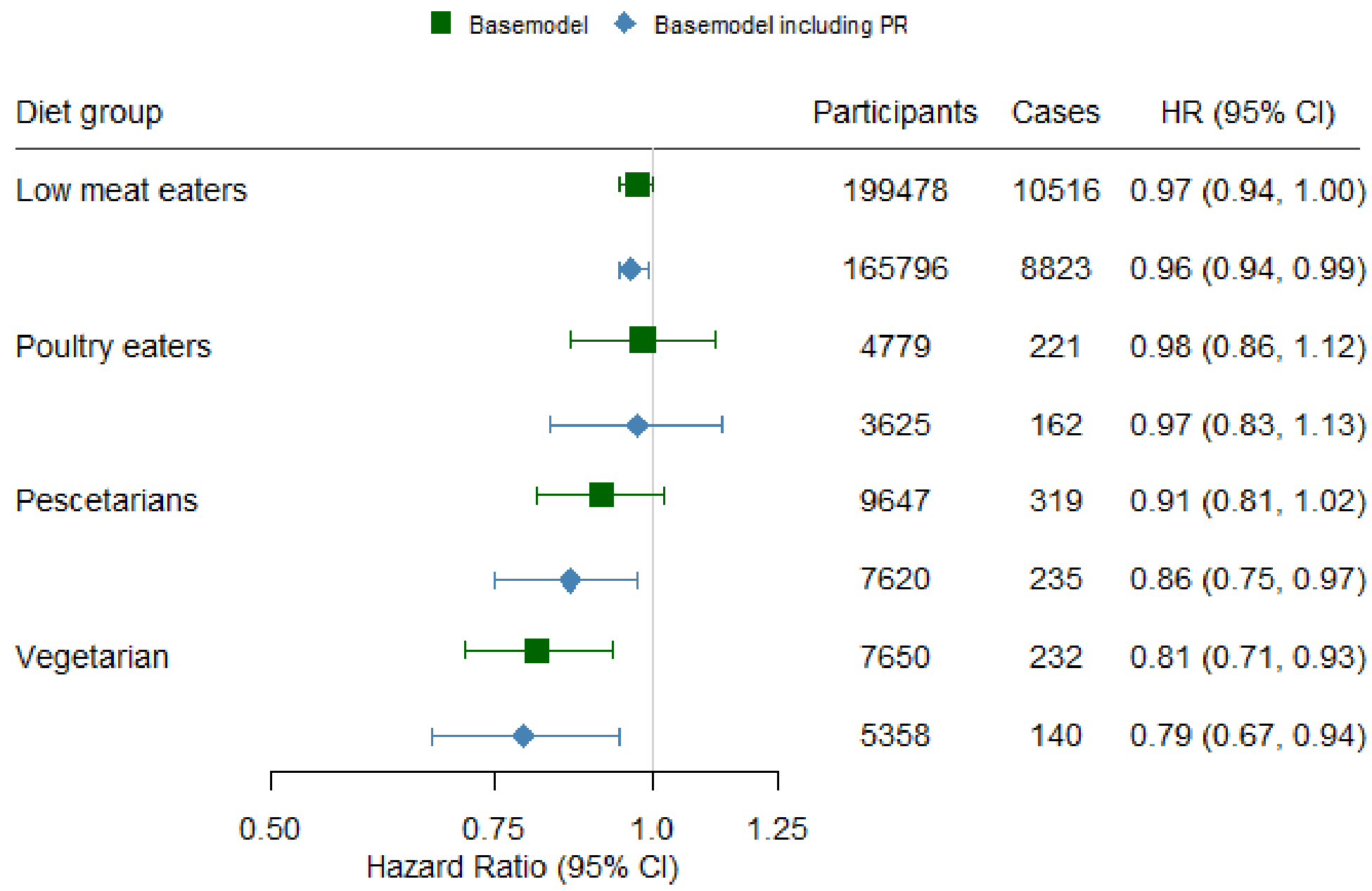

References

1. Levin A, Stevens PE, Bilous RW, et al. Kidney Disease: Improving Global Outcomes (KDIGO) CKD Work Group. KDIGO 2012 clinical practice guideline for the evaluation and management of chronic kidney disease. *Kidney international supplements*. 2013;3(1):1-150.
2. *kidney.epi: Kidney-Related Functions for Clinical and Epidemiological Research*. 2025. Accessed 04/08/2025. https://Scientific-Tools.Org
3. Thompson AS, Gaggl M, Bondonno NP, et al. Adherence to a healthful plant-based diet and risk of mortality among individuals with chronic kidney disease: A prospective cohort study. *Clin Nutr*. Oct 2024;43(10):2448-2457. doi:10.1016/j.clnu.2024.09.021
